# Free intraperitoneal tumor cells and outcome in gastric cancer patients: a systematic review and meta-analysis

**DOI:** 10.18632/oncotarget.5595

**Published:** 2015-09-10

**Authors:** Mathieu Pecqueux, Johannes Fritzmann, Mariam Adamu, Kristian Thorlund, Christoph Kahlert, Christoph ReiΔfelder, Jürgen Weitz, Nuh N. Rahbari

**Affiliations:** ^1^ Department for Visceral, Thoracic and Vascular Surgery, University of Dresden, Dresden, Germany; ^2^ Department of Clinical Epidemiology & Biostatistics, McMaster University, Hamilton, Ontario, Canada

**Keywords:** free intraperitoneal tumor cells, peritoneal lavage, gastric cancer, prognosis

## Abstract

**Purpose:**

Despite continuously improving therapies, gastric cancer still shows poor survival in locally advanced stages with local recurrence rates of up to 50% and peritoneal recurrence rates of 17% after curative surgery. We performed a systematic review with meta-analyses to clarify whether positive intraperitoneal cytology (IPC) indicates a high risk of disease recurrence and poor overall survival in gastric cancer.

**Methods:**

Multiple databases were searched in December 2014 to identify studies on the prognostic significance of positive intraperitoneal cytology in gastric cancer, including: Medline, Biosis, Science Citation Index, Embase, CCMed and publisher databases. Hazard ratios (HR) and associated 95% confidence intervals (CI) were extracted from the identified studies. A meta-analysis was performed using a random-effects model on overall survival, disease-free survival and peritoneal recurrence free survival.

**Results:**

A total of 64 studies with a cumulative sample size of 12,883 patients were included. Cytology, quantitative real time polymerase chain reaction (PCR) or both were performed in 35; 21 and 8 studies, respectively. Meta analyses revealed free intraperitoneal tumor cells (FITC) to be associated with poor overall survival in univariate (HR 3.27; 95% CI 2.82 - 3.78]) and multivariate (HR 2.45; 95% CI 2.04 - 2.94) analysis and poor peritoneal recurrence free survival in univariate (4.15; 95% CI 3.10 - 5.57) and multivariate (3.09; 95% CI 2.02 - 4.71) analysis. Subgroup analysis showed this effect to be independent of the detection method, Western or Asian origin or the time of publication.

**Conclusions:**

FITC oder positive peritoneal cytology is associated with poor survival and increased peritoneal recurrence in gastric cancer.

## INTRODUCTION

Every year around one million new cases of gastric cancer are diagnosed globally. In 2012, 723,000 people died from gastric cancer, ranking it the 4^th^ most common cancer-related cause of death. Complete surgical resection together with perioperative chemotherapy represents the standard of care for curative treatment of patients with gastric cancer [1-3]. However, even after multimodal therapy up to 40% of the patients experience disease recurrence and up to 30% die within 12 months [4].

Peritoneal dissemination is a common cause of failure after curative treatment for gastric cancer. Peritoneal recurrence occurs in 17% of patients undergoing resection with curative intent and is associated with a dismal survival [5, 6]. Due to the frequent occurrence and the strong prognostic relevance of peritoneal metastases, detection of free intraperitoneal tumor cells (FITC) has been suggested as a prognostic and predictive biomarker in gastric cancer patients [7, 8]. Detection of FITC may help to recognize those patients considered for curative therapy who are at high-risk for early tumor relapse and might benefit from intensified treatments such as hyperthermic intraperitoneal chemotherapy (HIPEC) [9]. Numerous studies have so far been conducted on the prognostic and predictive value of FITC in gastric cancer. Although FITC are found in 6-49% of gastric cancer patients considered for curative surgery [10-13], it's predictive and prognostic value has remained unclear due to inconsistent detection techniques and results of the individual studies. This clinical uncertainty is reflected by inconsistent recommendations made by different guidelines on the use of FITC in the management of gastric cancer [1-3, 14].

To clarify the role of intraperitoneal lavage cytology as a prognostic biomarker in gastric cancer, we performed a systematic review with meta-analyses of studies on the prognostic significance of FITC detection in peritoneal lavage samples of patients with gastric cancer considered for curative therapy.

## RESULTS

### Baseline study characteristics

**Figure 1:**
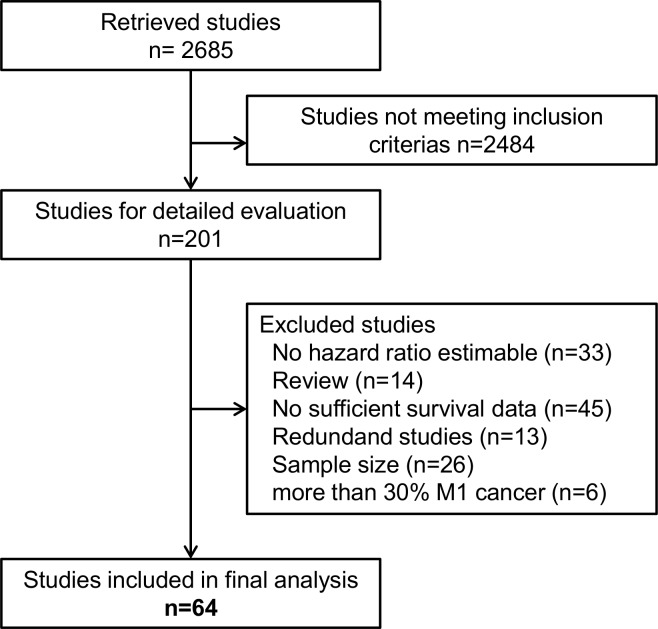
Flow diagram showing the selection process for relevant studies

In total, we included 64 studies [10-13, 15-68] with a cumulative sample size of 12, 883 patients (Figure 1). These studies had a median sample size of 134 (52 - 1297) patients and were published between 1978 and 2014 (Table 1). The included studies were conducted in Western institutions in 19% and in Asian institutions in 81%. Patients with stage IV disease were enrolled in 30 (47%) studies. The median follow up across all studies was 35 (18 - 82) months. FITC were detected by cytology in 43 (67%) studies (38 studies used Papanicolaou staining, 5 studies used H&E staining), by immunocytochemistry (ICC) in 5 (8%) studies and by RT-PCR in 29 (45%) studies (Table 2). The majority of studies used Carcinoembryonic antigen (CEA) for molecular tumor cell detection. In 22 studies CEA expression was analyzed and in seven studies CK20 expression was analyzed. Further markers included CK19, CD44, Caspase 9, MINT, MAGE, MMP 7, CA125, TGFβ, RegIV, FABP1, Muc2, IL-17 and CDH1. The detection rate of FITC across the included studies varied markedly (median: 23%; range 6% - 58%) and showed a strong association with patients’ stage of disease and in particular the inclusion of patients with overt peritoneal metastases. FITC were detected prior to resection in 62 (97%) studies and pre- as well as postoperativelv in 2 (3%) studies. OS, DSS, DFS and PRFS was reported in 51 (80%), 7 (11%), 11 (17%) and 21 (33%) studies, respectively. Hazard ratios for multivariate analysis could be extracted in 21 studies (ten that performed cytology, eight that performed RT-PCR and three that performed both). Fifteen studies were graded with a low risk of bias (Appendix 1). Funnel plot analyses did not indicate significant publication bias for the analyzed outcomes (Appendix 2).

**Table 1:** Baseline characteristics of included studies

First author	Year of publication	Study type	Period of enrollment	Country of origin	Sample size	Age	Female/Male (%)	Stage	M1	FITC positive (%)	Neo. Chem. (%)	Adj. Chem. (%)	Pall. Chem.	Chemotherapy Regimen
Badgwell, B	2008	RSCS	1995 - 2005	USA	379	61	35%/65%	UICC II-IV	22%	15%	6%	NA	26%	NA
Bando	1999	RSCS	1975 - 1997	Japan	1297	NA	NA	UICC II-IV	23%	24%	NA	NA	NA	NA
Benevolo	1998	RSCS	1989 - 1996	Italy	80	61	41%/59%	UICC I-III	0%	15%	0%	50%	NA	NA
Bentrem	2005	RSCS	1993 - 2002	USA	371	57	44%/56%	UICC I-III	0%	6%	NA	NA	NA	NA
Boku	1990	RSCS	1984 - 1987	Japan	93	NA	NA	T4, P0	0%	20%	NA	NA	NA	NA
Bonenkamp	1996	RSCS	1989 - 1993	Netherland	457	30% >70	41%/59%	UICC I-III	0%	7%	NA	NA	NA	NA
Chang Qing	2011	RSCS	2006 - 2007	China	53	54	57%/43%	UICC I-IV	NA	58%	0%	NA	NA	NA
Chuwa,	2005	PSCS	1998 - 2002	Singapore	142	67	36%/64%	UICC I-IV	25%	25%	0%	NA	NA	NA
Euanorasetr, C.	2007	RSCS	1997 - 2005	Thailand	97	59	50%/50%	UICC I-III	0%	23%	NA	NA	NA	NA
Fujimoto	2002	RSCS	1981-1997	Japan	96	60	32%/68%	UICC I-III	0%	33%	0%	13%	NA	5FU; MMC; OK-432
Fujiwara	2014	PSCS	2007 - 2009	Japan	137	66	32%/68%	UICC I-IV	8%	CY 20%; PCR 61%	0%	71%	NA	S1
Fukagawa	2010	RSCS	1992 - 1998	Japan	573	NA	NA	UICC II-IV	18%	28%	0%	58%	NA	NA
Fukuda, Y	2011	RSCS	2001 - 2009	Japan	71	69	25%/75%	T4a + b M0	0%	38%	0%	71%	NA	5-FU; Paclitaxel
Han	2014	RSCS	2008 - 2009	China	92	74% <60	41%/59%	UICC I-IV	8%	49%	0%	NA	NA	NA
Hao, Y. X.	2010	PSCS	2004 - 2009	China	164	53	59%/41 %	UICC I-III	0%	55%	0%	NA	NA	NA
Hara	2007	RSCS	2001 - 2003	Japan	76	63	67%/33%	UICC I-III	0%	15%	0%	NA	NA	NA
Hayes	1999	RSCS	1992 - 1994	UK	85	69	22%/78%	UICC I-IV	24%	19%	NA	NA	NA	NA
Horikawa	2011	RSCS	2000 - 2006	Japan	147	66	66%/34%	UICC II-III	0%	33%	NA	NA	NA	NA
Iida	2013	RSCS	2003 - 2006	Japan	79	35% >65J	27%/73%	UICC I-III	0%	44%	0%	NA	NA	NA
Ikeguchi	1994	RSCS	1976 - 1989	Japan	229	60	39%/61%	UICC II-IV	23%	33%	0%	100%	NA	NA
Ishii	2004	RSCS	1999 - 2002	Japan	60	NA	NA	UICC I-IV	15%	23%	0%	NA	NA	NA
Ito	2005	PSCS	2000-2002	Japan	283	NA	NA	UICC I-III	0%	23%	0%	21%	NA	NA
Jeon	2010	RSCS	2002 - 2003	Korea	84	44% >65	38%/62%	UICC I-III	0%	13%	NA	NA	NA	NA
Jeon	2014	RSCS	2009 - 2010	Korea	117	45% >65	42%/58%	UICC I-III	0%	33%	0%	NA	NA	NA
Jiang	2011	RSCS	1997 - 2002	China	139	57	32%/68%	UICC I-IV	28%	27%	0%	27%	22%	NA
Kang	2014	PSCS	2010 - 2010	Korea	75	41% >65	33%/67%	UICC I-III	0%	9%	NA	NA	NA	5FU
Katsuragi	2007	RSCS	NA	Japan	117	NA	NA	UICCI-IV	11%	19%	NA	NA	NA	NA
Kodera	1998	RSCS	NA	Japan	148	NA	NA	UICC I-IV	17%	CY 16%; PCR 28%	0%	NA	NA	NA
Kodera	1999	PSCS	1995 - 1998	Japan	91	61	60%/40%	UICC II-III	0%	11%	0%	26%	NA	NA
Kodera	2006	RSCS	1995 - 1999	Japan	274	60	NA	UICC I-IV	12%	38%	0%	NA	NA	NA
La Torre	2010	PSCS	2003 - 52008	Italy	64		NA	UICC I - III	0%	11%	0%	42%	NA	NA
Lee	2012	RSCS	2001 - 2009	Korea	1072	55	33%/67%	UICC I-IV	14%	16%	NA	69%	NA	NA
Li	2005	PSCS	1995 - 1997	China	64	59	34%/66%	UICC I-III	0%	23%	0%	NA	NA	NA
Li	2014	RSCS	2007 - 2008	China	116	61	39%/67%	UICC I-III	0%	35%	0%	61%	NA	NA
Makino	2010	RSCS	2002 - 2006	Japan	113	63	37%/63%	UICC II-IV	10%	31%	0%	30%	NA	MMC + Cisplatin
Manzoni	2006	RSCS	1992 - 2002	Italy	168	65	37%/63%	UICC I-III	0%	14%	0%	0%	NA	NA
Miyagawa	2008	RSCS	1999 - 2004	Japan	95	NA	32%/68%	UICC I-IV	16%	49%	NA	NA	NA	NA
Miyashiro	2005	RSCS	1975 - 1994	Japan	320	61	39%/61%	UICC I-III	0%	8%	NA	92%	NA	NA
Nakajima	1978	RSCS	1972 - 1976	Japan	196	NA	NA	UICC I-III	0%	16%	0%	43%*; 58%**	NA	MMC + 5FU or Futraful + Cytarabine
Nekarda	1999	PSCS	1987 - 1990	Germany	118	59	33%/67%	UICC I-III	0%	20%	0%	0	NA	NA
Oyama	2004	RSCS	1997 - 2001	Japan	163	64	32%/68%	UICC I-IV	14%	28%	0%	17%*; 93%**	NA	5-FU +/− Cisplatin
Ozer	2012	RSCS	2000 - 2007	Turkey	255	60	35%/65%	UICC I-III	0%	14%	NA	NA	NA	NA
Ribeiro	2006	PSCS	1993 - 2002	Brazil	201	61	36/64%	UICC I-III	0%	7%	0%	0%	NA	NA
Rosenberg	2006	RSCS	1987 - 2001	Germany	346	64	37%63%	UICC I-III	0%	21%	0%	NA	NA	NA
Ryu	2008	RSCS	2001 - 2006	Korea	424	60	36%/64%	UICC I-IV	22%	27%	NA	NA	NA	NA
Satoh	2012	RSCS	NA	Japan	61	NA	NA	UICC I-IV	26%	23%	NA	NA	NA	NA
Sugita	2003	RSCS	1998 - 2002	Japan	114	NA	NA	UICC I-IV	10%	CY 7%; PCR 46%	0%	NA	NA	NA
Suzuki	1999	RSCS	1988 - 1996	Japan	347	NA	NA	UICC I-IV	4%	8%	NA	8,4%	NA	NA
Takata	2013	RSCS	2009 - 2012	Japan	104	63% >65J	46%/54%	UICC I-III	0%	15%	19%	NA	NA	S1 + Cisplatin and/or Docetaxel
Takebayashi	2014	RSCS	2009 - 2012	Japan	102	68	42%/58%	UICC I-III	0%	56%	NA	NA	NA	NA
Tamura	2007	RSCS	2000 - 2005	Japan	164	NA	35%/65%	UICC I-IV	9%	27%	NA	NA	NA	NA
Tamura	2014	PSCS	2007 - 2009	Japan	124	66	30%/70%	UICC I-IV	10%	CY 20%; PCR 61%	0%	71%	NA	S1
Tokuda	2003	RSCS	1997 - 1999	Japan	131	NA	32%/68%	UICC I-III	0%	CY 4%; PCR 22%	NA	NA	NA	NA
Ueno	2003	RSCS	1998 - 2001	Japan	79	NA	33%/67%	UICC I-IV P0	5%	40%	0%	0%*; 100%**	NA	NA
Vogel	1999	PSCS	1992 - 1995	Germany	75	65	34%/66%	UICC I-III	0%	42%	NA	NA	NA	NA
Wong	2012	PSCS	2007 - 2009	USA	118	NA	NA	UICC I-IV	0%	20%	NA	NA	98% ***	NA
Wu	1997	RSCS	1990 - 1993	Taiwan	129	64	21%/79%	UICC II-III	0%	19%	0%	NA	NA	MMC
Yamamoto	2009	RSCS	2000 - 2006	Japan	566	NA	34%/66%	UICC I-IV	20%	10%	0%	98% ***	NA	S1 or S1 combination
Yamamoto	2014	RSCS	2006 - 2011	Japan	193	68.4	NA	UICC I-IV	21%	27%	NA	21%	NA	S1
Yamashita	2009	RSCS	1990 - 2000	Japan	232	NA	31%/69%	UICC I-III	0%	34%	NA	NA	NA	NA
Yoneda	2014	RSCS	2007 - 2008	Japan	52	68	33%/67%	UICC I-IV	23%	40%	NA	NA	NA	NA
Yonemura	2001	RSCS	1993 - 1999	Japan	230	59	20%/80%	UICC I-III	0%	CY 19%; PCR 17%	NA	NA	NA	NA
Yoshikhawa	2003	RSCS	1987 - 1997	Japan	149	61	33%/67%	UICC II-III	0%	22%	NA	NA	NA	NA
Yu	2012	RSCS	2008 - 2009	China	92	74% <60	41%/59%	UICC I-IV	8%	49%	0%	NA	NA	NA

Legend:

Study type: Prospective cohort study (PSCS) and retrospective cohort study (RSCS)

Age: Age includes median age. If median age was not available, mean age was used.

Stage: Included stages. All stages are calculated according to the official TNM classification of the “Union internationale contre le cancer” (UICC) [1]

M1: Percentage of patients that had metastasized disease (M1)

FITC positive: Percentage of patients that had free intraperitoneal tumor cells (FITC)

Neo. Chem.: Percentage of patients that received neoadjuvant chemotherapy

Adj. Chem.: Percentage of patients that received adjuvant chemotherapy

Pall. Chem.: Percentage of patients that received palliative chemotherapy

Chemotherapy Regimen: Includes all used chemotherapy regimens in the study; used abbreviations: Fluorouracil (5FU); Mitomycin C (MMC), Picibanil (OK-432), oral fluoropyrimidine S-1 (S-1)

1. Sobin LH, Gospodarowicz MK, Wittekind C. TNM Classification of Malignant Tumours. John Wiley & Sons; 2011. 209 p.

**Table 2:** Design variables of included studies

First author	Year of publication	A/W	CY	ICC	PCR	sample size	detection (rel. to surgery)	Sample quantity (ml)	collection site **	detection target	LAD	Median follow up (median and range)	Outcomes reported	Multivariate
Badgwell	2008	W	1			379	before surgery	1000	PC	CY	NA	51	OS	not significant
Bando	1999	A	1			1196	before surgery	200	D, BO, LS	CY	NA	NA	OS; PRFS	significant
Benevolo	1998	W	1	1		80	before surgery	50	PC	CY	NA	>24 (24 - NA)	OS	not performed
Bentrem	2005	W	1				before surgery	100	RS, LS, D	CY	NA	36	OS	significant
Boku	1990	A	1			93	before surgery	100	D	CY	NA	NA	OS	not performed
Bonenkamp	1996	W	1			535	before surgery	200	D	CY	D1 - D2	NA	OS	not performed
Chang Qing	2011	A			1	53	before surgery	NA	Ascites	Caspase 9	NA	NA	OS	not performed
Chuwa	2005	A	1			138	before surgery	200	D, LS, LPG	CY	D2	36 (13 to 59)	OS; DFS	significant
Euanorasetr, C.	2007	A	1			97	before surgery	100	D, BO, LS, RPG	CY	D2	49 (3-119)	OS	significant
Fujimoto	2002	A	1			236	before surgery	200	D, LS	CY	NA	>36 (36 - NA)	OS	significant
Fujiwara	2014	A	1		1	137	before surgery	100	PC	CEA	D2	NA, probably 60	OS; PRFS; DFS	significant
Fukagawa	2010	A	1	1		701	before surgery	100	D	CY	NA	NA	OS	not tested
Fukuda	2011	A	1			71	before surgery	NA	NA	CY	D2	24 (1-89)	OS	significant
Han	2014	A			1	92	before surgery	200	D, RS, LS, LPG	MINT2	NA	NA	DFS	not significant
Hao, Y. X.	2010	A	1		1	164	before and after surgery	100	D	CEA	D2	38 (3–63)	OS	not tested
Hara	2007	A	1		1	76	before surgery	100	D, LPG	CEA CK 20	NA	22 (4.6 - 43)	OS; DFS	significant
Hayes	1999	W	1			85	before surgery	100	IT, LPG, D	CY	D2	24	OS	not tested
Horikawa	2011	A			1	147	before surgery	100	D	CD 44	NA	37 (7-68)	OS; PRFS	significant
Iida	2013	A			1	79	before surgery	100	D	IL-17	NA	61	OS	significant
Ikeguchi	1994	A	1			229	before surgery	50	D	CY	NA	48 - 216	OS	not performed
Ishii	2004	A	1		1	60	before surgery	200	D, LS	CEA	NA	NA	OS; PRFS	not performed
ito	2005	A			1	86	before surgery	200	D, LS	CEA	NA	30 (21 - 50)	OS; PRFS	significant
Jeon	2010	A			1	84	before surgery	200	D	MAGE	NA	60	OS	significant
Jeon	2014	A			1	117	before surgery	100	D	MAGE/CEA	NA	36 (NA)	DFS	significant
Jiang	2011	A	1			139	before surgery	100	D, LS	CY	NA	>60	OS	significant
Kang	2014	A	1			75	before surgery	400	D, LS	CY	NA	30	OS; DFS	not performed
Katsuragi	2007	A			1	116	before surgery	100	D, LS	CEA; CK20	D2	32	OS	significant
Kodera	1998	A			1	148	before surgery	100	D	CEA	D2	18 (5 - 32)	OS; PRFS	not performed
Kodera	1999	A	1			91	before surgery	100	D, LS	CY	D2/D3	25 (11 - 45)	OS; PRFS	significant
Kodera	2006	A	1		1	274	before surgery	100	D	CEA	D2	82 (60 - 142)	OS; PRFS	significant
La Torre	2010	W	1	1		64	before surgery	200	D, LS, RS, LPG	CEA	D1 + CT	32 (12 - 56)	OS	significant
**Lee**	2012	A	1			**1072**	before surgery	200	D, BO, LS, RS	CY	D2	NA	OS	significant
Li	2005	A	1			64	before surgery	50	RS or D	CY	NA	39 (9-74)	OS	only significant vor PRFS
Li	2014	A			1	116	before surgery	100	D	*	NA	36	PRFS	significant
Makino	2010	A	1			113	before surgery	500	PC	CY	NA	29	OS	not tested
Manzoni	2006	W	1			168	before surgery	200	IT, D	CY	D2	64 (35 - 159	OS; PRFS; DFS	not significant
Myiagawa	2008	A			1	95	before surgery	150	D	CY	NA	>24	OS	significant
Miyashiro	2005	A	1			320	before surgery	150	D	CY	NA	NA	OS	not tested
Nakajima	1978	A	1			274	before surgery	200	IT, RS, LS	CY	NA	NA	OS	not tested
Nekarda	1999	W	1	1		118	before surgery	500	RS, LS	CY	D2	69 (41 - 84)	OS	significant
Oyama	2004	A			1	195	before surgery	100	D	CEA	D0 – D2	26 (1,4 - 51)	OS; PRFS; DFS	significant
Ozer	2012	W	1			255	before surgery	50	LS	CY	D2-D3	18 (0,2 - 107)	OS	not significant
Ribeiro	2006	A	1			201	before surgery	100	IT, LS, RS	CY	D2	64 (55 - 73)	OS	significant
Rosenberg	2006	W	1	1		346	before surgery	500	IT, LS	Ber-EP4	NA	70 (24 - 204)	OS	significant
Ryu	2008	A	1			424	before surgery	200	IT, D	CY	NA	24	OS; PRFS	significant
Satoh	2012	A			1	61	before surgery	NA	NA	CY	NA	24 (1 - 33)	PRFS	not performed
Sugita	2003	A	1		1	123	before surgery	100	D, LS	CEA/CK20	NA	NA	OS; PRFS; DFS	not performed
Suzuki	1999	A	1			347	before surgery	100	D, LS	CY	NA	NA	OS	significant
Takata	2013	A			1	104	before surgery	100	D, LS	CEA, CK-20	NA	18	DFS	significant
Takebayashi	2014	A			1	102	before and after surgery	100	PC	CEA/CK20	NA	NA	PRFS	not performed
Tamura	2007	A				164	before surgery	100	D, LS	CEA/CK20	NA	26 (18 - 65)	OS; PRFS	significant
Tamura	2014	A				137	before surgery	100	D, LS	CEA/CK20	D2	NA, probably 60	PRFS	significant
Tokuda	2003	A	1			136	before surgery	200	D, LS	CY	NA	27 (17 - 39)	OS; PRFS	not performed
Ueno	2003	A	1		1	124	before surgery	100	D, LS	CEA	D2	30 (3 - 50)	OS	not performed
Vogel	1999	W	1			75	before surgery	100	IT	CY	NA	45 (24.7 - 66)	OS	not performed
Wong	2012	W			1	118	before surgery	NA	LS, RS, D	CEA	NA	35	OS	not performed
Wu	1997	A	1			129	before surgery	200	IT, LS	CY	D2	NA	OS	not performed
Yamamoto	2009	A	1			566	before surgery	100	D	CY	NA	30 (12 - 96)	OS	significant
Yamamoto	2014	A			1	193	before surgery	100	D	CEA/CA 72-4	D2	32	OS	significant
Yamashita	2009	A	1			232	before surgery	NA	NA	CY	NA	>5y	OS	significant
Yoneda	2014	A			1	52	before surgery	400	PC	CK19	NA	39 (6 - 51)	OS; PRFS; DFS	not performed
Yonemura	2001	A	1		1	230	before surgery	1000	PC	CY	NA	41 (4 - 74)	OS; PRFS	significant
Yoshikawa	2003	A	1			149	before surgery	100	D, LS	CY	D2/D3	NA	OS; PRFS	significant
Yu	2012	A			1	92	before surgery	200	RS, LS, LPG	meth.CDH1	NA	NA	OS	significant

Legend:

A/W: Study population originating from asian (A) or western (W) countries

CY/ICC/PCR: Shows which detection method was used to detect free intraperitoneal cells (FITC): cytology (CY), Immunocytochemistry (ICC) or quantitative real time polymerase chain reaction (PCR). 1 means the paper includes results using the mentioned detection method.

Collection site: BO = bursa omentalis; D = douglas pouch; LS = left subphrenic space, RS = right subphrenic space; LPG = left paracolic gutter; RPG = right paracolic gutter; PC = peritoneal cavity (not specified)

Detection target: only cytology (CY), Carcinoembryonic antigen (CEA), MINT2 gene (MINT2), cytokeratin (CK) 20, melanoma associated antigen (MAGE), Cluster of Differentiation (CD)-44, Interleukin (IL)-17, Ber-EP4 antibody (Ber-EP4), Carbohydrate Antigen (CA) 72-4, CK 19, methylated Cadherin-1 (CDH1), Matrix-Metalloproteinase (MMP)-7, Transforming growth factor (TGF)-b, * = CEA, MMP 7, CK 20, CA 125, TGF b

Lymphadenectomy (LAD): information not available (NA), D1, D2, coeliac trunc (CT)

Outcomes reported: Overall survival (OS), disease free survival (DFS), peritoneal recurrence free survival (PRFS)

### Prognostic value of FITC detection

**Figure 2:**
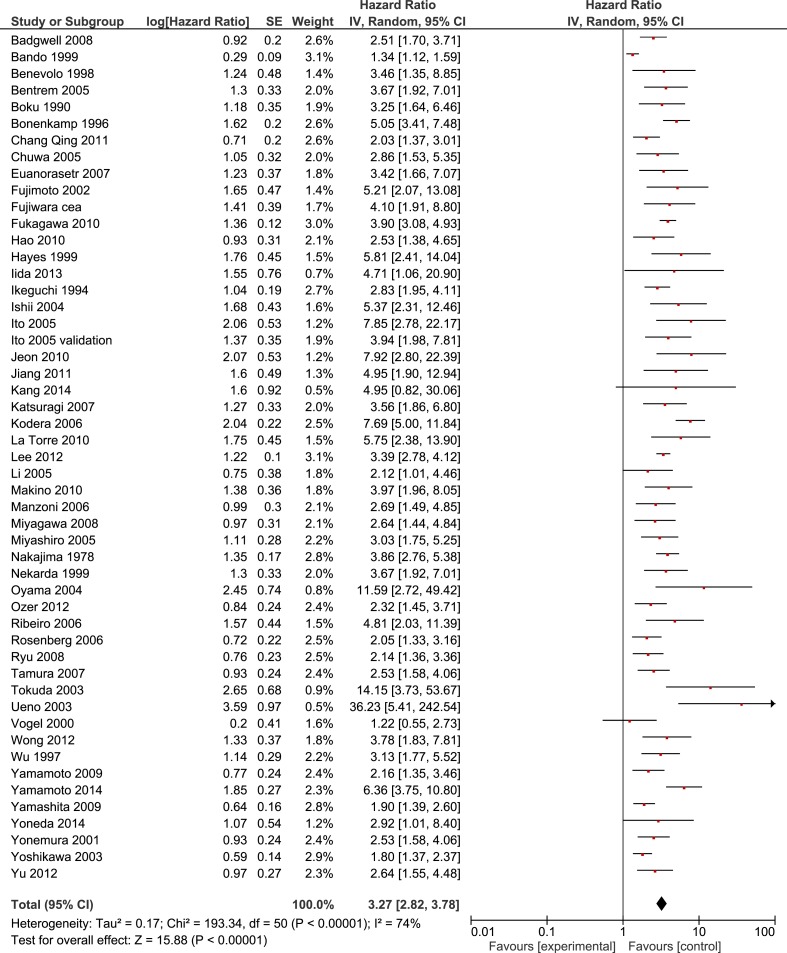
Forest plot for the prognostic value of FITC in patients with gastric cancer (Overall survival)

Some 51 studies with a cumulative sample size of 11, 005 patients reported on OS.^10-13, 23-32, 34, 36, 38-43, 46-49, 51-60, 63-65, 67-7^ The pooled analyses of the results from these studies showed a strong prognostic value of FITC detection (HR 3.27, 95% CI 2.82 - 3.78; *n* = 51; I² = 74%) (Figure 2). This result could be verified in the 35 studies with curatively resected patients and a cumulative sample size of 5908 (3.51; 3.01 - 4.08; *n* = 35; I^2^ = 48%) (Table 3) [10-13, 16-19, 22, 24, 25, 30-35, 37, 41, 44-49, 51, 56, 59, 60, 62, 65, 66, 68, 69]. Sensitivity analyses failed to identify a single study as a reason for the observed statistical heterogeneity. Meta-analysis of the results from 17 studies with multivariate analyses confirmed the prognostic association of FITC detection with reduced heterogeneity (2.45; 2.04 - 2.94; *n* = 17; I² = 39%) [11, 12, 21, 23, 25, 32, 33, 38, 40, 48, 52, 54, 62, 63, 65, 66, 68]. Furthermore, we found significant associations of FITC detection and long-term outcome in the pooled analyses on DFS (3.61; 2.63 - 4.96; *n* = 11; I² = 26%)[21, 23, 27, 34, 44, 48, 53, 64, 70, 71] and PRFS (4.15, 3.10 - 5.57; *n* = 14; I² = 30%) (Table 4) [12, 23, 31, 38, 42, 44, 55, 56, 64-66, 72, 73].

**Table 3:** Subgroup analyses for overall survival in FITC positive patients and curatively resected FITC positive patients

	Overall Survival					Overall Survival (Curative)				
	HR	95% CI	Heterogeneity I^2^ (%)	P-value	Included Studies	HR	95% CI	Heterogeneity I^2^ (%)	P-value	Included Studies
**Total:**	3.27	2.82 - 3.78	74%	0.00001	51	3.51	3.01 - 4.08	48%	0.00001	35
**Multivariate:**	2.45	2.04 - 2.94	39%	0.00001	17	3.37	2.04 - 5.57	65%	0.00001	8
**Detection Method:**										
*CY*	3.03	2.55 - 3.61	78%	0.00001	35	3.19	2.75 - 3.69	38%	0.00001	25
*PCR*	3.64	2.93 - 4.53	49%	0.00001	19	5.07	3.50 - 7.36	53%	0.00001	13
**Stage of disease**										
*Advanced stage of disease*	2.88	2.47 - 3.36	0%	0.00001	19	2.52	2.10 - 3.02	24%	0.00001	12
*All stages*	3.58	3.07 - 4.17	45%	0.00001	34	3.23	2,98 - 3.50	41%	0.00001	27
**Date of publication**										
*up to and including 2005*	3.49	2.68 - 4.56	80%	0.00001	23	3.61	2.98 - 4.37	45%	0.00001	21
*after 2005*	3.13	2.66 - 3.68	64%	0.00001	28	3.39	2.60 - 4.40	54%	0.00001	14
**Study population**										
*Asian*	3.31	2.77 - 3.95	78%	0.00001	38	3.64	3.04 - 4.36	43%	0.00001	24
*Western*	3.17	2.50 - 4.01	48%	0.00001	13	2.9	2.16 - 3.90	53%	0.00001	9
**Size of study population**										
*<median*	3.62	2.99 - 4.39	34%	0.00001	26	3.77	2.80 - 5.09	51%	0.00001	17
*>median*	2.98	2.45 - 3.62	84%	0.00001	25	3.25	2.74 - 3.84	43%	0.00001	16
**Risk of bias**										
*high*	3.08	2.62 - 3.62	74%	0.00001	39	3.35	2.85 - 3.95	42%	0.00001	26
*low*	3.96	2.92 - 5.38	63%	0.00001	12	4.27	2.89 - 6.33	62%	0.00001	9
**Lavage fluid**										
*>150ml*	3.38	2.47 - 4.27	80%	0.00001	23	3.7	2.93 - 4.68	49%	0.00001	15
*<150ml*	3.36	2.75 - 4.10	65%	0.00001	25	3.47	2.78 - 4.32	52%	0.00001	18
**FITC positive (%)**										
*>median*	3.31	2.61 - 4.19	81%	0.00001	28	3.36	2.67 - 4.21	56%	0.00001	20
*<=median*	3.15	2.72 - 3.64	46%	0.00001	24	3.73	3.07 - 4.53	31%	0.00001	15
**Chemotherapy**										
*>25% adj. Chemo*	3.56	3.15 - 4.01	0%	0.00001	9	3.56	2.86 - 4.43	42%	0.00001	7
*<25% adj. Chemo*	4.51	3.21 - 6.35	52%	0.00001	11	4.25	3.13 - 5.79	31%	0.00001	9
*no adj. Chemo*	4.37	2.30 - 8.29	57%	0.00001	4	3.1	2.00 - 4.78	0%	0.00001	2
*no neoadj. Chemo*	3.48	2.96 - 4.10	50%	0.00001	27	3.55	2.98 - 4.22	35%	0.00001	19

Legend: Legend: HR: Hazard Ratio; 95% CI: 95% confidence interval; CY = cytology; PCR = polymerase chain reaction

**Table 4:** Subgroup analyses for disease free survival (DFS) and peritoneal recurrence free survival (PRFS) in FITC positive patients

	DFS					PRFS				
	HR	95% CI	Heterogeneity I^2^ (%)	P-value	Included Studies	HR	95% CI	Heterogeneity I^2^ (%)	P-value	Included Studies
**Total:**	3.61	2.63 - 4.96	26%	0.00001	11	4.15	3.10 - 5.57	30%	0.00001	14
**Multivariate:**	7.26	2.95 - 17.88	52%	0.00001	3	3.09	2.02 - 4.71	65%	0.00001	9
**Detection Method:**										
*CY*	4.37	2.22 - 8.60	38%	0.00001	4	4.94	3.27 - 7.47	15%	0.00001	8
*PCR*	3.42	2.32 - 5.03	28%	0.00001	7	4.06	2.86 - 5.76	0.36	0.00001	10
**Stage of disease**										
*curative*	4.21	2.59 - 6.86	43%	0.00001	7	5.07	3.28 - 7.82	0.55	0.00001	11
*Advanced*	9	3.60 - 22.54	0%	0.00001	2	3.21	1.85 - 5.58	56%	0.0001	5
*not advanced*	3.38	2.50 - 4.56	17%	0.0009	10	4.63	3.43 - 6.24	15%	0.00001	12
**Date of publication**										
*up to and including 2005*	4.49	1.98 - 10.2	53%	0.0003	3	6.21	3.43 - 11.25	36%	0.00001	5
*after 2005*	3.41	2.40 - 4.85	21%	0.00001	8	3.4	2.54 - 4.55	9%	0.00001	9
**Study population**										
*Asian*	3.88	2.71 - 5.56	29%	0.00001	10	4.14	3.02 - 5.67	34%	0.00001	13
*Western*	2.66	1.48 - 4.80	NA	0.001	1	5.05	1.93 - 13.20	NA	0.0009	1
**Size of study population**										
*<median*	4.82	2.73 - 8.52	21%	0.00001	5	5.28	3.14 - 8.88	31%	0.00001	7
*>median*	3.08	2.16 - 4.38	18%	0.00001	6	3.58	2.56 - 5.00	25%	0.00001	7
Risk of bias										
*high*	2.99	2.27 - 3.95	0%	0.00001	8	3.7	2.58 - 5.30	23%	0.00001	9
*low*	8.52	4.13 - 17.59	0.6	0.00001	3	5	3.00 - 8.35	42%	0.00001	5
**Lavage fluid**										
*>150ml*	3.5	2.44 - 5.03	0%	0.00001	5	5.22	3.42 - 7.98	17%	0.00001	7
*<150ml*	4.13	2.31 - 7.40	52%	0.00001	6	3.41	2.29 - 5.07	36%	0.00001	7
**Cytology positive patients**										
*>median*	3.77	2.44 - 5.83	26%	0.00001	5	3.27	2.06 - 5.17	42%	0.00001	5
*<=median*	3.61	2.16 - 6.04	37%	0.00001	6	4.58	3.25 - 6.45	0%	0.00001	8
**Chemotherapy**										
*>25% adj. Chemo*	2.29	1.25 - 4.21	NA	0.007	1	3	1.55 - 5.8	52%	0.001	2
*<25% adj. Chemo*	3.67	2.29 - 5.89	22%	0.00001	3	5.45	3.14 - 9.46	6%	0.00001	3
*no adj. Chemo*	3.26	2.12 - 5.00	0%	0.00001	2	5.05	1.93 - 13.2	NA	0.0009	1
*no neoadj. Chemo*	3.99	2.62 - 6.06	47%	0.00001	8	4.67	3.28 - 6.66	0%	0.00001	6

Legend: HR: Hazard Ratio; 95% CI: 95% confidence interval; CY = cytology; PCR = polymerase chain reaction

### Subgroup analyses

Subgroup analyses were performed to assess the impact of the detection method on the results. These analyses revealed a prognostic association of FITC detection by cytology with OS (3.03; 2.55 - 3.61; *n* = 35; I² = 78%) [10, 11, 13, 15-19, 21, 22, 24, 25, 28, 30, 33, 34, 38-41, 43, 44, 46, 47, 49-52, 57, 60, 61, 63, 65, 66, 69]. Despite a lower number of studies we observed a more pronounced prognostic value for pooled analyses of studies using RT-PCR (3.64; 2.93 - 4.53; *n* = 19; I² = 49%) [12, 20, 23, 26, 35, 38, 42, 45, 48, 53, 55-57, 59, 62, 64, 65, 67, 68]. This difference reached statistical significance in the test of interaction for the subgroup of patients who underwent potentially curative resection (*p* = 0.012). The kind of detection method had no impact on the prognostic value with respect to DFS and PRFS (Table 3, Table 4).

We next evaluated the prognostic value of FITC in patients with advanced stages as compared to the entire patient cohort. Only one study reported outcome selectively for patients with early stage of disease (without lymph node metastases) [51]. There was a significant association of FITC detection with OS in patients with advanced disease as well as the entire cohort. However, in particular for patients who underwent a potentially curative resection, the magnitude of effect was lower in case of advanced disease (2.52; 2.10 - 3.02; *n* = 12; I² = 24%)[16, 18, 25, 27, 30, 36, 47, 51, 59, 60, 65, 66] than for studies including the entire population (3.23; 2.98 - 3.50; *n* = 27; I² = 41%)[10-13, 17, 19, 22, 24, 31-35, 41, 45, 46, 48, 49, 51, 56, 59, 62, 65, 68, 69] (*p* = 0.014; test of interaction). The increased prognostic value of FITC detection in patients with less advanced disease was confirmed for PRFS (*p* = 0.008, test of interaction). There was not enough data for a pooled analysis of advanced disease for DFS (*n* = 2).

Previous studies suggested genetic differences between gastric cancers dependent on geographic location [74-76]. We therefore evaluated the prognostic value of FITC detection separately for these cohorts. These analyses showed a significant association between FITC detection and OS for Asian population (3.31; 2.77 - 3.95; *n* = 38; I² = 78%) [11-13, 16, 18, 20-22, 24, 26, 30-35, 38, 40, 41, 43, 45, 46, 48, 52, 55-57, 60-68] as well as Western population (3.17; 2.50 - 4.01; *n* = 13; I² = 48%) [10, 15, 17, 19, 28, 39, 44, 47, 49-51, 59, 69]. Significant associations for both cohorts were also present for patients who underwent a curative resection as well as the outcomes DFS and PRFS with no significant difference between both population as indicated by the tests of interaction.

Systemic chemotherapy has become common practice in the curative therapy of advanced gastric cancer [1, 77, 78], though the optimal regimen is still subject to intensive research [77]. Previous studies showed that 60-90% of FITC positive patients can be converted to FITC negative by neoadjuvant chemotherapy and thus improve survival [79, 80]. We therefore evaluated the prognostic value of FITC depending on the administration of neoadjuvant and adjuvant chemotherapy, respectively. These analyses revealed a strong association of FITC detection and OS, DFS and PRFS independent of the administration of neoadjuvant or adjuvant chemotherapy.

To exclude that the observed results were primarily caused by studies with low methodological quality, further analyses were stratified for the risk of bias. While studies with low (3.96; 2.92 - 5.38; *n* = 12; I² = 63%)[11, 28, 38, 39, 47, 48, 51, 55, 65] and high risk of bias (3.08; 2.62 - 3.62; *n* = 39; I² = 74%)[10, 13, 15-24, 26, 30-34, 40, 41, 43-46, 49, 50, 52, 56, 57, 59-64, 66-69] showed a significant prognostic value for FITC detection on OS, the effect was more pronounced in studies with low risk of bias (*p* = 0.15; test of interaction). The enhanced prognostic value reported in studies with a low risk of bias supports the validity of the finding that FITC detection represents a strong prognostic marker in gastric cancer.

## DISCUSSION

This systematic review and meta-analysis shows a marked association of FITC with overall survival, disease free survival and peritoneal recurrence free survival of patients with gastric cancer scheduled for curative therapy.

Although the first studies on detection of FITC in gastric cancer patients have been published over 60 years ago [81], the role of FITC detection in the management of patients with gastric cancer has remained highly controversial. This may in part be explained by different study designs and insufficient statistical power of individual studies, in particular for subpopulations of patients with different extent of disease. In line with this, current gastric cancer treatment guidelines do not provide uniform recommendations on the use of peritoneal lavage. Although the majority of guidelines classify FITC detection as metastatic (M1) disease, these recommendations are based on single or a few individual studies, are limited to peritoneal lavage cytology and do not provide any standardization with respect to the sampling time and sampling/detection methodology (i.e. amount of lavage fluid, kind of staining). While the NCCN guidelines recommend a staging laparoscopy with peritoneal washings for cytology for stage IB and higher, the European ESMO, ESSO, ESTRO guidelines are less stringent and recommend a staging laparoscopy with or without peritoneal washings for malignant cells in these patients [1, 2]. Furthermore, there is no consensus regarding the consequences of a positive peritoneal cytology on patients’ clinical management. In the NCCN guidelines a positive peritoneal cytology is considered a criterion of unresectability for cure. The European guidelines do not comment on the consequences for surgical resection and the German guidelines state no relevance on patients’ further management [1, 2, 14]. As in these guidelines positive peritoneal cytology is classified as M1 disease and palliative treatment is recommended in M1 patients, there is urgent need to clarify which patients at what timepoint should undergo peritoneal lavage sampling by what methodology [26, 67, 73].

The results of the present meta-analysis confirm FITC as poor prognostic marker in patients with gastric cancer. Importantly, our results demonstrate the prognostic value of FITC detection to be dependent on the extent of disease. A more pronounced prognostic relevance is shown in patients with limited disease and a curative resection, respectively. Identification of strong prognostic markers might be useful in the management of gastric cancer patients in various ways. First, prognostic biomarkers might, moreover, serve as predictive biomarkers in patients considered for perioperative chemotherapy. Second, reliable prognostic information may be of particular help in decision-making for further treatment in elderly patients or patients with severe comorbidities who may be at increased risk for complications and poor outcome after multi-modal therapy. As total gastrectomy is associated with relevant morbidity and 90-day mortality, [82] a strong prognostic biomaker might be helpful to avoid surgery in high-risk patients with a poor prognosis. Third, it may be helpful in the management of young patients with excellent performance status who may be able to tolerate intensive therapy. Fourth, validation of FITC as strong prognostic biomarkers provide a valid scientific rationale for subsequent research to further characterize these cells on a molecular level. As targeted therapies are emerging for gastric cancer, [83] it is of particular interest, if molecular analysis of free intraperitoneal tumor cells might serve as a predictive biomarker for targeted agents in gastric cancer patients.

There is indeed increasing effort to identify patients with gastrointestinal malignancies and peritoneal metastases who benefit from intensified therapies such as HIPEC [84-86]. At present, these efforts mainly focus on patients with overt peritoneal metastases and showed promising results for colorectal cancer [87, 88]. The findings were much more modest for gastric cancer patients with overt peritoneal metastasis [89, 90] and may be explained by limitations to achieve complete cytoreduction [91]. These data suggest FITC positive gastric cancer without further distant metastasis as a promising subgroup of patients who might benefit from HIPEC. The first randomized controlled study to examine the benefit of extensive intraoperative peritoneal lavage followed by intraperitoneal chemotherapy in FITC positive gastric cancer showed promising results [92]. Further randomized controlled trials have already been initiated (ClinicalTrials.gov; NCT01683864). The results may redefine the treatment of FITC positive gastric cancer.

The optimal method of FITC detection remains to be determined. As outlined current guidelines are restricted to conventional cytology without providing further information on the kind of staining. Our results indicate a prognostic value of FITC detection by cytology as well as molecular techniques. To date, only few studies directly compared cytology by Papanicolaou staining with molecular detection by PCR [23, 29, 38, 53, 55-57, 59, 65]. Detection methods using PCR offer a considerably higher detection sensitivity at a marginally lower specificity (Appendix 3). This meta-analysis demonstrates a similar prognostic value for both detection methods. The results of the above studies imply a potential superiority of FITC detection by PCR, that needs to be substantiated within prospective trials before valid recommendations can be made in guidelines.

The use of peritoneal lavage in patients undergoing multimodal therapy remains a further question to be answered. While metabolic imaging has been proposed as a strategy for early response assessment in patients with cancers of the esophagogastric junction and stomach [93-95], peritoneal washings with detection of FITC may offer an additional or alternative approach. There is indeed evidence that clearance of positive peritoneal cytology by systemic chemotherapy is associated with improved outcome after surgical resection for gastric cancer [96, 97]. However, controlled clinical trials are required to clarify the benefit of surgical resection in patients who remain positive for FITC after chemotherapy.

**Figure 3:**
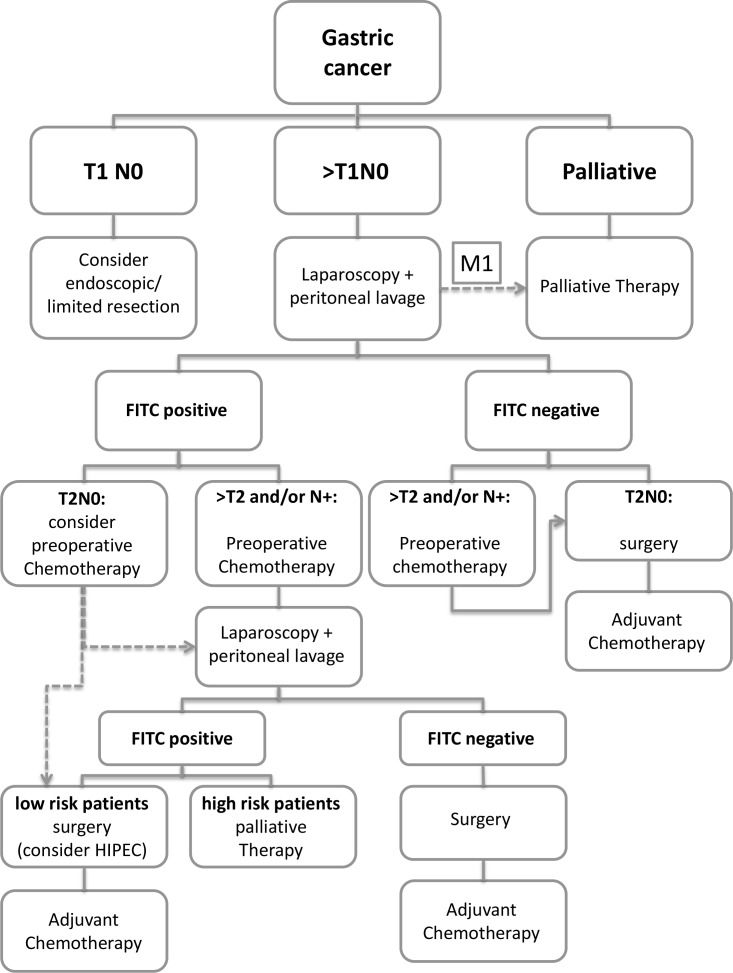
Treatment algorithm for gastric cancer

One important question that needs answering is how to proceed with FITC positive patients with potentially curative gastric cancer. Considering the results of this meta-analysis we would like to propose a therapeutic algorithm (Figure 3). However, the feasibility and clinical utility of this algorithm needs to be tested in controlled clinical trials.

In conclusion, this meta-analysis reveals FITC detection as poor prognostic marker in gastric cancer patients scheduled for curative therapy. The prognostic value of FITC was noted across detection methods, administration of chemotherapy and geographic location, though a more pronounced effect was observed in patients with less advanced disease. These results support efforts to use FITC as a predictive biomarker and may contribute to the development of uniform international treatment guidelines with the ultimate aim to improve individualized therapy and outcomes of patients with gastric cancer.

## MATERIALS AND METHODS

This systematic review was performed according to the recommendations of the PRISMA statement [98].

### Search strategy

A systematic search of the following databases was performed in December 2014: Medline, Science Citation Index, Embase, CCMed, Publisher Database, ASCO abstracts. Additionally, clinical trial registries such as WHO International Clinical Trials Registry and ClinicalTrials.gov were searched. Search strategies included the Medical Subject Headings (MeSH) “Stomach Neoplasm”; “Peritoneal Lavage”; “Therapeutic Irrigation”; “Cytology” as well as the text terms “gastric cancer”, “peritoneal”, “washing”, “lavage” and “cytology” in various combinations. In addition, we searched the reference lists of relevant articles and review articles. No time and language restrictions were applied to the initial search. The identified titles and abstracts were screened for eligibility by two independent reviewers (MP and MA). Full articles of potentially relevant studies were obtained for detailed evaluation.

### Study inclusion and exlcusion criteria

Studies were included based on predefined selection criteria. Studies were eligible for inclusion, if they included patients with histologically proven gastric cancer and investigated the association of FITC with at least one of the following time-to-event outcomes: Overall survival (OS: date of surgery to date of death of any cause); disease specific survival (DSS: date of surgery to date of death due to gastric cancer); disease free survival (DFS: date of surgery to date of recurrence or death of any cause, whichever comes first), recurrence free survival (RFS: date of surgery to date of recurrence) or peritoneal recurrence (PR: date of surgery to date of peritoneal recurrence). Peritoneal cytology may have included any standard staining technique (i.e. hematoxylin and eosin [H&E], Papanicolaou) performed on peritoneal fluid or peritoneal washings. Molecular detection methods may have included immunocytochemistry and any form of reverse-transcriptase polymerase chain reaction ([RT]-PCR). In contrast to DNA or protein markers, studies using peritoneal tumor mRNA markers were included, assuming a linear correlation between peritoneal tumor cell detection and extremely short-lived free mRNA molecules.

Exclusion criteria were met, if less than 50 peritoneal samples were analyzed, if the percentage of patients with peritoneal or distant metastasis was > 30%, if they were not published in a peer-reviewed journal, if the above mentioned definitions of peritoneal cytology or molecular diagnostic were not met or if no hazard ratio could be calculated for at least one of the above mentioned time-to-event outcomes.

### Data extraction

The following data was extracted from every article: first author, year of publication, study type, enrolment period, sample size, patient age and sex, FITC detection rate, definition of positive peritoneal fluid/lavage, timing of FITC detection, detection protocol, target genes and antigens, chemotherapy (neoadjuvant and/or adjuvant, treatment regimen), duration of follow up, reported outcomes and the use of multivariate models. The data for each included article were extracted independently by two authors (MP and MA). Diverging results were resolved by discussion.

### Assessment of study quality

Study quality was evaluated using the modified risk of bias tool recommended by the Cochrane Collaboration as described before [99, 100].

### Statistical analyses

The synchronized extraction results were pooled statistically as effect estimates in meta-analyses. Hazard ratios (HR) and their corresponding standard errors (SE) were extracted for the individual time-to-event outcome parameters of the included studies. In case the HR together with their associated SE or confidence intervals (CI) were not provided for a certain outcome, HRs were calculated using different statistical methods based on the clinical and statistical data reported in the primary studies [101, 102].

The extracted HR were pooled using the generic inverse variance method of the Review Manager Version 5.3 software (Copenhagen: The Nordic Cochrane Centre; The Cochrane Collaboration, 2014). To adjust for expected inter-study heterogeneity (study populations, treatments, detection assays, definitions of FITC positivity, duration of follow-up, etc.) a random effects analysis model was applied, which is more conservative when determining confidence intervals (CI) around the pooled HR [103]. I^2^ statistics was applied to assess the presence of statistical heterogeneity [104]. To explore reasons for statistical heterogeneity we performed sensitivity analyses, where the impact of single studies on the I^2^ value is tested as well as “a priori” subgroup analyses [105]. The results of subgroup analyses were compared by tests of interaction [105]. To avoid double patient evaluation among studies that evaluated multiple detection assays and/or target genes, these parameters were combined where possible to keep a maximum of information. Otherwise, cytokeratins were prioritized over alternative tumor cell markers and immunohistochemistry over RT-PCR assays. Sensitivity analyses (by choosing the alternative study arm) were performed to assess the statistical impact of such prioritization. Publication bias was assessed using funnel plot analyses.

## SUPPLEMENTARY MATERIAL


