# Supplementary material for: Free intraperitoneal tumor cells and outcome in gastric cancer patients: a systematic review and meta-analysis
Source: Oncotarget. 2015 Sep 10;6(34):35564–78. doi: 10.18632/oncotarget.5595 (PMC4742125; doi:10.18632/oncotarget.5595)
Supplement: Supplementary file 1 [file oncotarget-06-35564-s001.pdf]

Free intraperitoneal tumor cells and outcome in gastric cancer patients: a systematic review and meta-analysis

Supplementary Material

Appendix 1: Evaluation of bias

	Were adequate eligibility criteria developed and applied?	Was the measurement of both exposure and outcome adequate?	Was confounding adequately controlled for?	Was the follow-up complete and adequate in duration?	Are study reports free of suggestion of selective outcome reporting?	Was the study free of other problems that put it at a high risk of bias?	Risk of bias
Badgwell	yes	yes	yes	yes	no	yes	High
Bando	yes	yes	yes	unclear	no	no	High
Benevolo	yes	yes	no	unclear	yes	yes	High
Bentrem	yes	yes	yes	unclear	no	no	High
Boku	yes	yes	no	unclear	yes	yes	High
Bonenkamp	yes	yes	no	yes	yes	yes	High
Chang Qing	no	unclear	no	yes	no	yes	High
Chuwa	yes	yes	yes	yes	yes	yes	Low
Emoto	yes	yes	yes	yes	yes	yes	Low
Euanorasetr	yes	yes	yes	yes	yes	yes	Low
Fujimoto	yes	yes	no	yes	yes	no	High
Fujiwara	yes	yes	yes	unclear	yes	yes	High
Fukagawa	yes	yes	no	unclear	yes	yes	High
Fukuda	yes	unclear	unclear	unclear	yes	no	High
Han	yes	yes	unclear	unclear	yes	yes	High
Hao	yes	yes	no	yes	yes	no	High
Hara	yes	yes	no	yes	no	yes	High
Hayes	yes	yes	yes	yes	yes	yes	Low
Hioki	yes	yes	yes	yes	yes	yes	Low
Homma	yes	yes	yes	unclear	yes	yes	Low
Horikawa	yes	yes	yes	yes	yes	yes	Low
Iida	yes	yes	no	yes	no	yes	High
Ikeguchi	no	yes	no	unclear	no	yes	High
Ishii	yes	yes	no	yes	yes	yes	High
Ito	yes	yes	yes	yes	yes	yes	Low
Jeon 2010	unclear	yes	no	yes	unclear	no	high
Jeon 2014	yes	yes	yes	yes	yes	yes	Low
Jiang	yes	yes	no	yes	yes	no	High
Kang	yes	yes	yes	yes	yes	no	High
Katsuragi	yes	yes	yes	yes	yes	yes	Low
Kodera 1998	no	yes	no	no	yes	yes	High
Kodera 1999	yes	yes	unclear	yes	yes	yes	Low
Kodera 2001	yes	yes	no	yes	yes	no	High
Kodera 2006	yes	yes	yes	yes	yes	yes	Low
La Torre	yes	yes	unclear	yes	yes	unclear	Low
Lee	yes	yes	yes	yes	yes	yes	High
Li	yes	yes	unclear	yes	no	yes	High
Li 2014	yes	yes	yes	yes	no	unclear	High
Majima	no	yes	no	yes	no	yes	High
Manzoni, G.	no	yes	no	yes	no	yes	High
Makino	yes	yes	yes	yes	no	yes	High
Miyagawa	yes	yes	yes	yes	no	yes	High
Miyashiro	yes	yes	unclear	no	yes	unclear	High
Nakajima	no	unclear	no	unclear	no	unclear	High
Nekarda	yes	yes	yes	yes	yes	yes	Low
Oyama	yes	yes	yes	yes	yes	yes	Low
Ozer	yes	yes	yes	no	no	yes	High
Ribeiro	no	yes	yes	yes	no	yes	High
Rosenberg	yes	yes	yes	yes	yes	yes	Low
Ryu	no	yes	yes	yes	yes	no	High
Satoh	no	no	no	yes	no	yes	High
Schauer	yes	yes	unclear	yes	no	yes	High
Schott	no	yes	no	yes	yes	yes	High
Sugita	yes	no	no	yes	no	yes	High
Suzuki	no	yes	yes	unclear	no	no	High
Takata	yes	yes	yes	no	yes	no	High

Takebayashi	yes	yes	no	unclear	no	yes	High
Tamura 2007	yes	yes	yes	yes	yes	yes	Low
Tamura 2014	yes	yes	yes	yes	no	yes	High
Tokuda	no	yes	no	yes	no	yes	High
Ueno	yes	yes	no	no	yes	yes	High
Vogel	no	yes	no	yes	no	yes	High
Wong	yes	yes	no	yes	no	no	High
Wu	yes	yes	no	yes	yes	unclear	High
Yamagata	no	yes	no	no	no	yes	High
Yamamoto 2009	no	yes	yes	yes	no	yes	High
Yamamoto 2014	no	yes	yes	yes	no	yes	High
Yamashita 2009	yes	no	yess	yes	yes	yes	High
Yoneda	yes	yes	no	unclear	yes	yes	High
Yonemura 2001	yes	yes	yes	unclear	yes	yes	Low
Yonemura 2001	yes	yes	yes	yes	yes	yes	Low
Yonemura 2009	yes	yes	no	no	no	yes	High
Yoshikawa	yes	yes	yes	no	no	no	High
Yu	yes	yes	not possible	no	no	no	High

The quality of included studies was assessed using the modified Cochrane Collaboration's tool and the key criteria for the quality assessment of observational studies recommended by the Grading of Recommendations, Assessment, Development and Evaluation (GRADE) Working Group [1,2].

1. Higgins J, Green S. Cochrane Handbook for Systematic Reviews of Interventions [Internet]. Version 5.1.0 [updated March 2011]. The Cochrane Collaboration; 2011. Available from: www.cochrane-handbook.org

2. Schünemann H, Brożek J, Guyatt G, Oxman A. GRADE handbook for grading quality of evidence and strength of recommendations [Internet]. The GRADE Working Group, 2013.; 2013. Available from: www.guidelinedevelopment.org/handbook

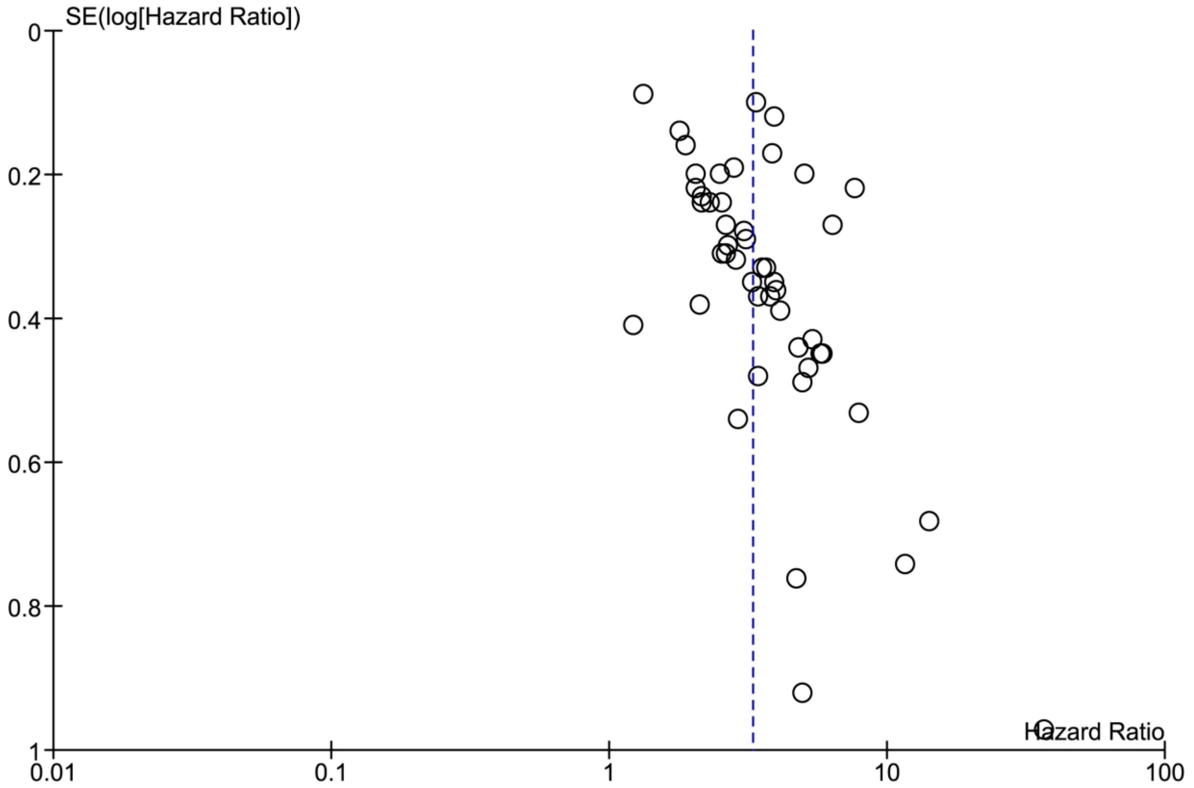
SE(log[Hazard Ratio])
0
0.2
0.4
0.6
0.8
1
0.01
0.1
1
10
100
Hazard Ratio

Appendix 3: Comparison of studies analyzing FITC detection by cytology and polymerase chain reaction.

First author	Year of publication	Sample size	Detection target	Stage	PCR Sensitivity	PCR Specificity	CY Sensitivity	CY Specificity
Hara	2007	76	CEA; CK20	UICC I-III	80%	82%	NA	NA
ito	2005	86	CEA	UICC I-III	85%	88%	NA	NA
Katsuragi	2007	116	CEA; CK20	UICC I-IV	81%	80%		NA
Kodera	2006	242	CEA	UICC I-IV	89%	82%	47%	97%
Sugita	2003	114	CEA; CK20	UICC I-IV	89%	63%	11%	93%
Tokuda	2003	131	CEA	UICC I-III	93%	88%	31%	100%
Ueno	2003	124	CEA	UICC I-IV P0	77%	68%	23%	98%
Yonemura	2001	230	CEA	UICC I-IV P0	57%	91%	46%	95%
				**Mean Value:**	**81%**	**80%**	**32%**	**97%**

1) Sensibility and specificity of FITC detection by cytology or polymerase chain reaction in the detection of peritoneal recurrence.

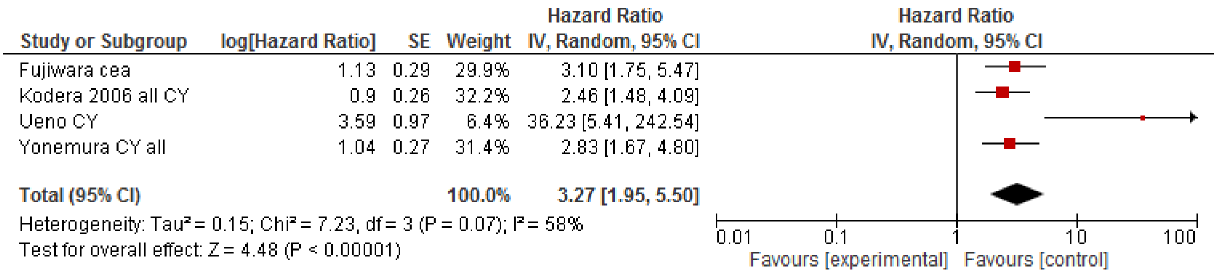

2) Hazard ratio of positive FITC detection by cytology

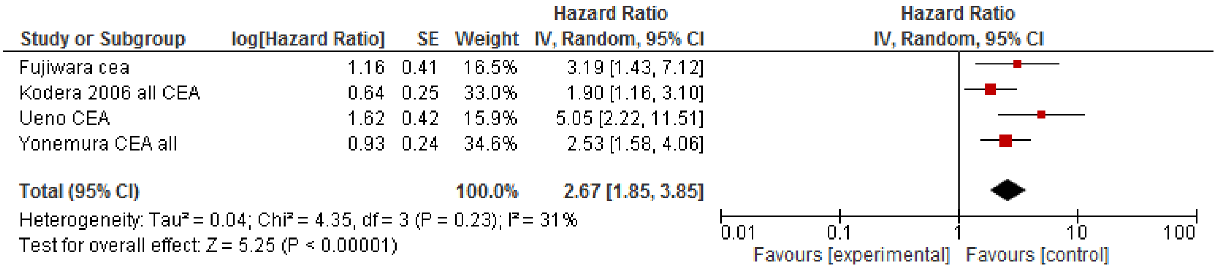

3) Hazard ratio of positive FITC detection by polymerase chain reaction

Study or Subgroup	log[Hazard Ratio]	SE	Weight	Hazard Ratio IV, Random, 95% CI
Fuijiwara CYo CEA	0.81	0.31	24.3%	2.25 [1.22, 4.13]
Horikawa CD44 all	0.77	0.23	44.1%	2.16 [1.38, 3.39]
Tamura all	1.2	0.31	24.3%	3.32 [1.81, 6.10]
Wong all	0.95	0.56	7.4%	2.59 [0.86, 7.75]
**Total (95% CI)**			**100.0%**	**2.45 [1.82, 3.31]**

Heterogeneity: Tau² = 0.00; Chi² = 1.35, df = 3 (P = 0.72); I² = 0%
Test for overall effect: Z = 5.88 (P < 0.00001)

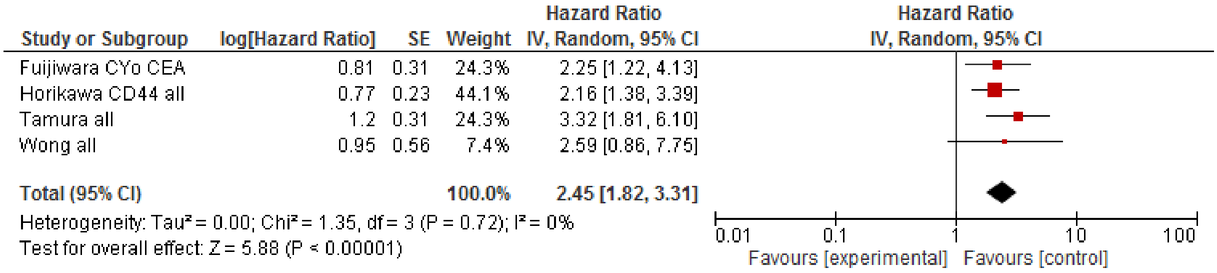

4) Hazard ratio of positive FITC detection by polymerase chain reaction in patients with negative cytology
